# Inflammatory Response during Neoadjuvant Chemotherapy with Immune Checkpoint Inhibitors in a Patient with T3N1 Triple-Negative Breast Cancer: A Case Report

**DOI:** 10.70352/scrj.cr.25-0172

**Published:** 2025-06-28

**Authors:** Kyoko Goda, Toshinari Yamashita, Mio Yasukawa, Takashi Yamanaka, Saori Fujiwara, Akari Takahashi, Emi Yoshioka, Yayoi Yamamoto

**Affiliations:** 1 Department of Breast Surgery and Oncology, Kanagawa Cancer Center, Yokohama, Kanagawa, Japan; 2 Department of Pathology, Kanagawa Cancer Center, Yokohama, Kanagawa, Japan; 3 Department of Radiology, Kanagawa Cancer Center, Yokohama, Kanagawa, Japan

**Keywords:** neoadjuvant chemotherapy, immune checkpoint inhibitors, inflammatory response, triple-negative breast cancer, pathological complete response, chemotherapy-induced inflammation

## Abstract

**INTRODUCTION:**

Preoperative chemotherapy, including immune checkpoint inhibitors (ICIs), is widely accepted as the most likely treatment regimen to obtain a pathological complete response (pCR) in triple-negative breast cancer (TNBC). This case report presents a rare instance of a pCR in a patient with cT3N1M0 TNBC who underwent neoadjuvant chemotherapy (NAC) with ICIs.

**CASE PRESENTATION:**

We present the case of a 44-year-old woman diagnosed with stage cT3N1M0 TNBC. The patient experienced a gradual enlargement of a left breast mass, axillary lymphadenopathy, and pain. Despite initial NAC with pembrolizumab, paclitaxel, and carboplatin, in addition to pembrolizumab, doxorubicin, and cyclophosphamide, tumor enlargement with an inflammatory response prompted surgical intervention. The patient underwent a left mastectomy with axillary lymph node dissection, resulting in a pCR with no viable tumor cells in the breast or lymph nodes. Postoperative radiotherapy and continued pembrolizumab therapy were administered. After 21 months of follow-up, the patient remains disease-free, with no evidence of recurrence or metastases.

**CONCLUSIONS:**

The patient’s inflammatory and cystic tumor response is an unreported variant of the NAC response, suggesting the potential for further exploration into the mechanisms driving such responses and their implications for treatment strategies.

## Abbreviations

CA
: cancer antigen
cCR
: clinical complete response
CEA
: carcinoembryonic antigen
CRP
: C-reactive protein
Hb
: hemoglobin
ICI
: immune checkpoint inhibitor
MMG
: mammography
NAC
: neoadjuvant chemotherapy
Neut
: neutrophils
pCR
: pathological complete response
PEM+AC
: doxorubicin+cyclophosphamide+pembrolizumab
PEM+CBDCA+PTX
: pembrolizumab+carboplatin+paclitaxel
Plt
: platelet
TNBC
: triple-negative breast cancer
US
: ultrasonography
WBC
: white blood cell

## INTRODUCTION

NAC combined with ICIs is considered a promising strategy for achieving pCR in patients with TNBC, particularly in locally advanced cases. This approach is particularly crucial for locally advanced cases, where achieving pCR is associated with improved long-term outcomes.^1,2)^

This case study presents a rare occurrence in which a patient with cT3N1M0 TNBC achieved pCR following NAC incorporating an ICI. The achievement of pCR in this setting underscores the potential of immunochemotherapy in downstaging tumors and improving prognosis, even in aggressive TNBC subtypes. This case contributes to the accumulating evidence supporting the role of ICIs in NAC, thereby reinforcing its clinical significance in optimizing treatment strategies for patients with TNBC.

## CASE PRESENTATION

A 44-year-old woman (156 cm, 81 kg) presented with a breast mass that had gradually increased over 1 year and was accompanied by axillary lymphadenopathy and breast pain noted 1 month prior to consultation with her primary physician. Cytological analysis of a 5 cm tumor beneath the left nipple revealed a Class V result, leading to a diagnosis of left breast cancer. She was referred to our institution for further evaluation and treatment. The patient had no significant past medical history. Her family history revealed that her father had rectal cancer.

**Fig. 1:**
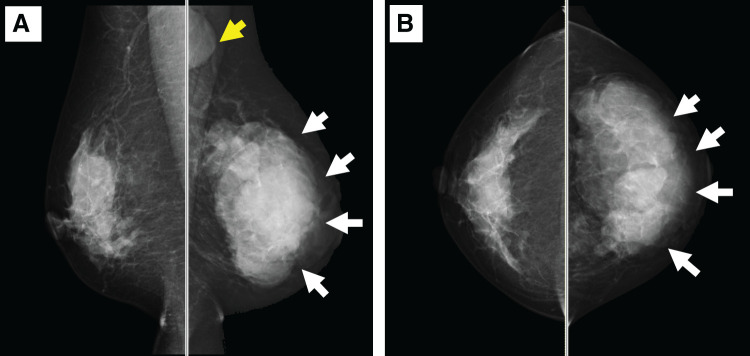
Initial MMG. (**A**) and (**B**) show the mediolateral oblique and craniocaudal views of the right breast MMG. The mass, indicated by white arrows, is characterized as a high-density with irregular, spiculated borders in the entire left breast with mild skin thickening. An axillary lymph node swelling is marked by a yellow arrow. These findings are the first indication of the presence of a malignant tumor and lymph node metastasis. MMG, mammography

**Fig. 2:**
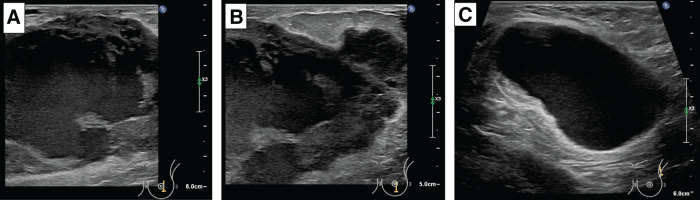
Initial US. Internal heterogeneity of the tumor and the presence of halo findings are noted. (**A**) Central portion of the left breast tumor, (**B**) caudal marginal portion of the left breast tumor, (**C**) swelling of axillary lymph nodes. These findings are the first indication of the presence of a malignant tumor with a mixed internal component and lymph node metastasis. US, ultrasonography

**Fig. 3:**
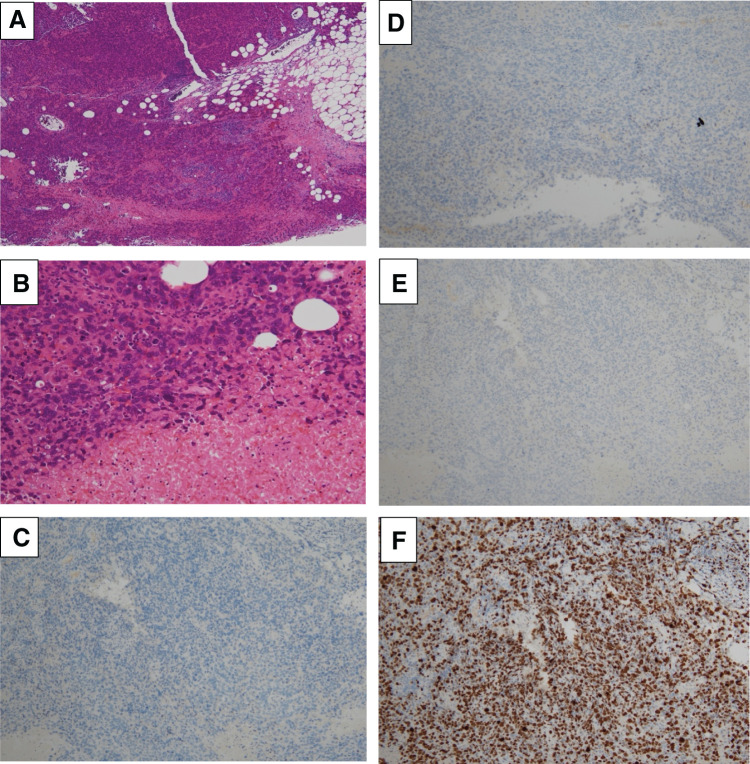
Histopathological findings from VAB. (**A**, **B**) HE stained at ×40, ×200 magnifications. (**C**–**F**) Immunohistochemical staining results are negative for estrogen and progesterone receptors, with a human epidermal growth factor-2 score of 0. The Ki-67 proliferation index is markedly high at 90%. A tumor-infiltrating lymphocytes score of 10%–20% was observed. These are findings of high-grade triple-negative breast cancer. HE, hematoxylin and eosin; VAB, vacuum-assisted biopsy

On examination, palpation revealed a 5 cm mobile tumor in the left breast without skin changes, erythema, or warmth. A soft, enlarged lymph node was palpable in the left axilla. No masses were detected in the contralateral breast. MMG revealed a high-density mass with irregular, spiculated borders in the left breast, accompanied by mild skin thickening (**Fig. 1**). US showed a well-demarcated, coarse, oval, heterogeneous mass extending beyond the measurable range, with blood flow and halo in the left breast, and a single axillary lymph node was swollen (**Fig. 2**). The histopathological findings from the US-guided vacuum-assisted biopsy revealed an invasive carcinoma characterized by solid proliferation and infiltrative clusters of atypical cells with papillary structures. The tumor was classified as nuclear grade 3, histological grade 3, estrogen receptor 0%, progesterone receptor 0%, human epidermal growth factor receptor-2: 0 (immunohistochemistry score), Ki-67: >20% (hot spot: 90%), and tumor-infiltrating lymphocytes score: 10%–20% (**Fig. 3**).^3)^

**Fig. 4:**
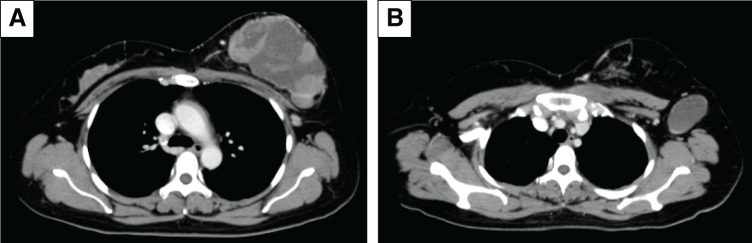
Contrast-enhanced CT. (**A**) The left breast tumor is composed of both liquid and solid components that are contrasted. (**B**) Cystic changes are observed in the axillary lymph nodes. These findings suggest a mixed internal tumor and lymph node metastasis.

**Fig. 5:**
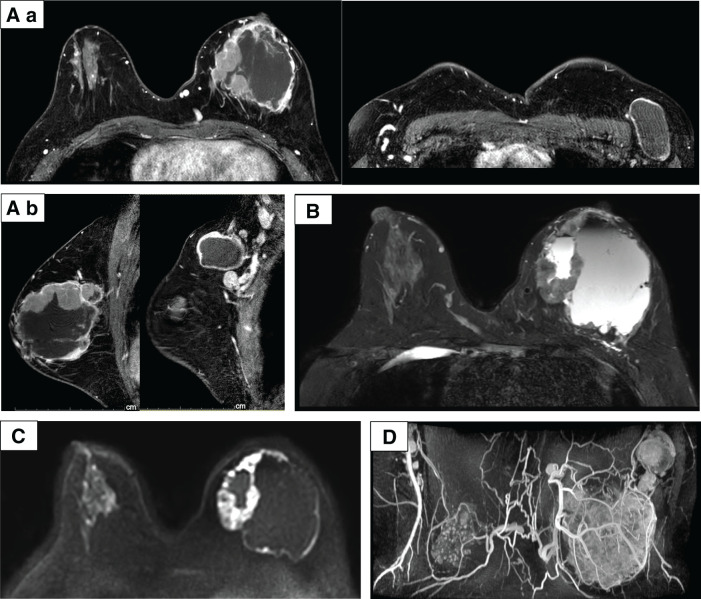
Contrast-enhanced MRI. (**A**) T1 (enhanced), rapid phase: (**a**) horizontal section and (**b**) sagittal section. (**B**) T2, (**C**) DWI, and (**D**) MIP. These findings suggest a mixed internal tumor, skin edema, and lymph node metastasis. DWI, diffusion-weighted imaging; MIP, maximum intensity projection

Furthermore, CT confirmed the 11.5 × 7.5 cm tumor in the left breast and a 5 cm axillary lymph node. No distant metastases were found (**Fig. 4**). MRI showed an 8.7 × 8.0 × 8.5 cm cystic tumor with several solid components exhibiting diffusion restriction and fast-plateau enhancement, in addition to wall invasion. The axillary lymph node exhibited cystic changes (**Fig. 5**).

Blood tests revealed no elevated tumor markers and normal WBC counts or inflammatory reactions: WBC count 4900/μL (Neut: 3040/μL), Hb 11.9g/dL, Plts 331000/μL, CRP <0.03 mg/dL, HbA1c 5.3%, CEA 1.2 ng/mL, and CA 15–3 5.5 U/mL.

The patient was diagnosed with cT3N1M0 TNBC based on clinical and histopathological findings. NAC was initiated according to the KEYNOTE-522 regimen, consisting of pembrolizumab in combination with chemotherapy: 1) initial chemotherapy with pembrolizumab 200 mg, paclitaxel 145 mg (80 mg/m^2^), and carboplatin 225 mg (area under the curve 1.5) (PEM+CBDCA+PTX); and 2) subsequent chemotherapy with doxorubicin 108 mg (60 mg/m^2^), cyclophosphamide 1080 mg (600 mg/m^2^), and pembrolizumab (PEM+AC).

**Fig. 6:**
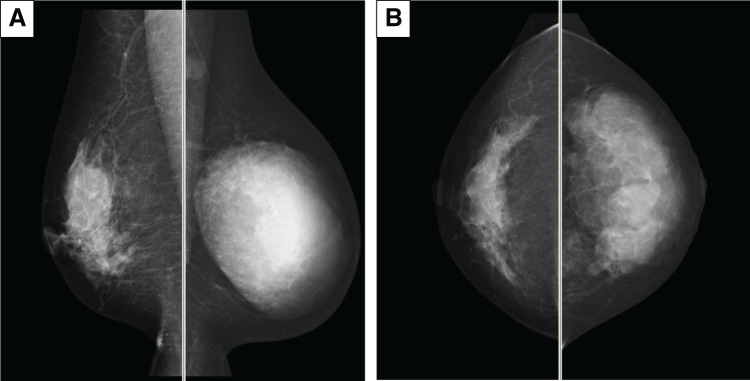
MMG at NAC midpoint (**A**: MLO right, left; **B**: CC right, left). A reduction in tumor and lymph node size is observed. CC, craniocaudal; MLO, mediolateral oblique; MMG, mammography; NAC, neoadjuvant chemotherapy

**Fig. 7:**
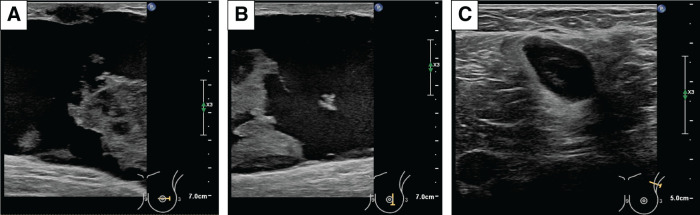
US at NAC midpoint (**A**: central portion of left breast tumor; **B**: caudal marginal portion of left breast tumor; **C**: Cystic changes of axillary lymph nodes). Tumor and lymph node size reduction is noted. NAC, neoadjuvant chemotherapy; US, ultrasonography

The following side effects were observed during the 1st half of preoperative chemotherapy. Peripheral neuropathy (grade 2) was observed in cycle #4-2, leading to a dose reduction of paclitaxel. Neutropenia requiring a 1-week delay occurred on cycles #3-1, #4-1, and #4-3. After 3 months, the breast tumor showed a slight reduction in size, and the axillary lymph node had decreased in size and softened, which was associated with pain relief (**Figs. 6** and **7**).

**Fig. 8:**
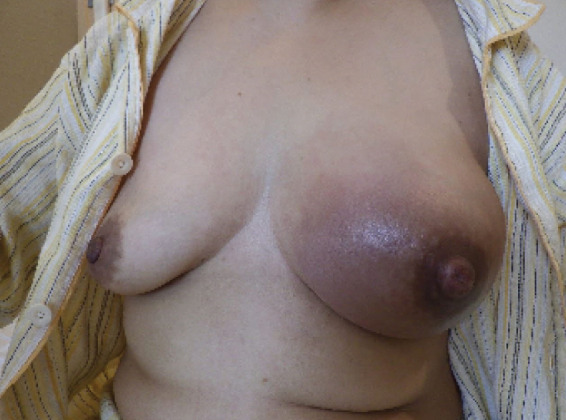
The image captured on cycle 2, day 23 provides a visual representation of the breast. The onset of pain was noted 5 days ago and has since worsened. Clinical findings indicate inflammatory changes in the breast tumor.

In the 2nd half of preoperative chemotherapy, 2 cycles were completed without significant adverse events. In the 3rd cycle of PEM+AC, rapid tumor enlargement, pain, erythema, and edema of the left breast were reported. Upon examination, a 10 cm mass was observed with redness, skin edema, and warmth with severe pain. The axillary lymph node had reduced to 2 cm. This symptom appeared in cycle 2, day 18, and worsened daily (**Fig. 8**).

**Fig. 9:**
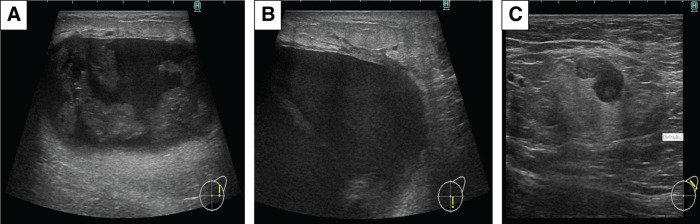
US after PEM+AC, 2nd cycle, at the time the patient reported rapid tumor growth and pain (**A**: inside the left external superior portion of the tumor; **B**: edematous changes in the subcutaneous tissue; **C**: axillary lymph node). These findings suggest inflammatory changes in the breast tumor. PEM+AC, doxorubicin+cyclophosphamide+pembrolizumab; US, ultrasonography

The US tumor findings remained the same, with a mixture of solid and liquid components. A thickened epidermis and elevated echogenicity of subcutaneous tissue appeared, and cystic changes of the axillary lymph nodes showed a tendency to shrink (**Fig. 9**).

**Fig. 10:**
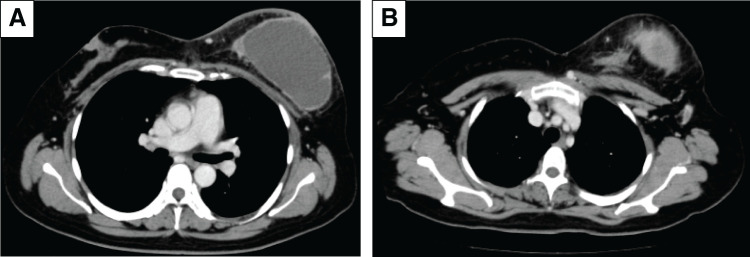
Contrast-enhanced CT captured on cycle 2 day 22 (**A**: left breast tumor with skin edema; **B**: shrunken axillary lymph node). These findings suggest inflammatory changes in the breast tumor.

**Fig. 11:**
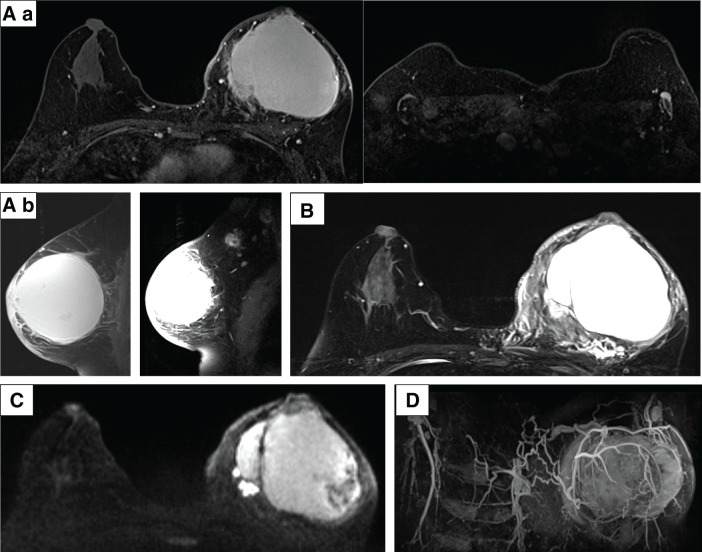
Contrast-enhanced MRI captured on cycle 2, day 22. (**A**) T1 (enhanced) rapid phase: (**a**) horizontal section (breast tumor, axillary lymph nodes) and (**b**) sagittal section (left: breast tumor, skin edema; right: breast tumor, axillary lymph nodes). (**B**) T2, (**C**) DWI, and (**D**) MIP. These findings suggest inflammatory changes in the breast tumor. DWI, diffusion-weighted imaging; MIP, maximum intensity projection

CT and MRI confirmed a predominantly cystic tumor with peripheral enhancement, skin thickening, and surrounding tissue edema. The axillary lymph nodes showed a further reduction without fluid retention (**Figs. 10** and **11**).

Blood tests showed a mildly elevated inflammatory response: WBC 8400/μL (Neut 6550/μL) and CRP 6.94 mg/dL.

The urin analysis results were negative for urine occult blood and leukocytes. Furthermore, the patient tested negative for the following viruses: influenza and SARS-CoV-2.

The clinical and imaging findings suggested 2 possibilities: 1) tumor enlargement with inflammatory response; and 2) tumor necrosis–induced inflammation. Given the severity of symptoms and poor response to conservative management (loxoprofen 180 mg/day and acetaminophen as needed), NAC was discontinued, and surgery was planned. The mammary skin was red and hot with edema. On clinical examination, it was possible to distinguish areas where the mass was near the skin with little mobility and areas where the mass was separated from the skin with mobility.

Despite the marked edematous change in the skin, it was uniform throughout, and there was no obvious evidence of subcutaneous nodules or enlarged lymph nodes on imaging. We thus considered the possibility of skin changes due to tumor necrosis-induced inflammation to be more likely than tumor enlargement with an inflammatory response. Therefore, in the surgery, the skin was designed as much as possible for mastectomy, and the skin incision line was designed over the range of mobility of the tumor, without setting the skin to an excessive extent that would require skin grafting. It was decided to administer antibiotics preoperatively and intraoperatively as in a normal breast cancer surgery, and to consider additional antibiotic administration based on the postoperative course. In consultation with the anesthesiologist, the patient was diagnosed with inflammation limited to the breast, and it was determined that preoperative anti-inflammatory treatment with steroids was unlikely to be necessary, and the patient's postoperative course was considered. In addition, since anthracyclines and taxanes, which are considered key drugs in preoperative chemotherapy for breast cancer, had been administered as preoperative chemotherapy, and surgery was possible at this point, continuation of chemotherapy with a drug change was not recommended, and the patient preferred surgery and did not wish to continue chemotherapy.

In May 20XX, the patient underwent a left mastectomy with axillary lymph node dissection. The patient did not develop fever or recurrent inflammatory findings and remained stable postoperatively.

**Fig. 12:**
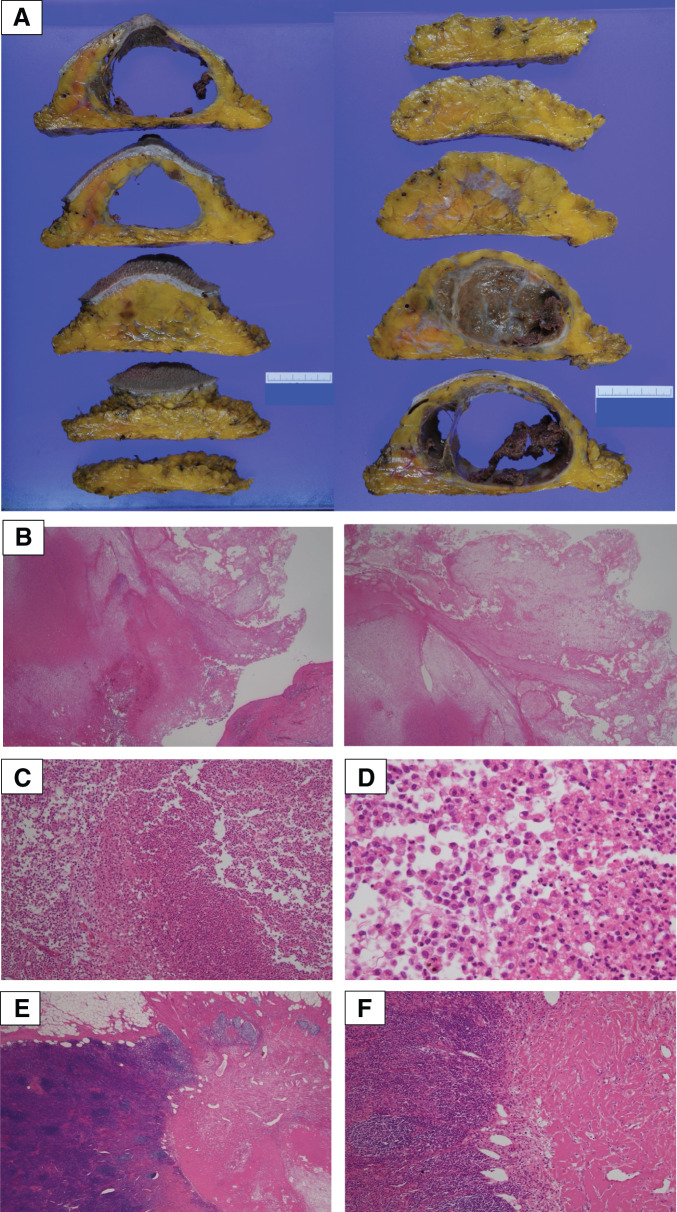
Histopathological findings from surgery: pCR. (**A**) Macroscopic findings of breast. (**B**) Breast mass findings, HE stained at ×20 magnification (left: the cyst wall and degeneration; right: degeneration in the cyst). (**C**, **D**) Breast mass findings, HE stained at ×100, 400 magnification: histiocytes and inflammatory cells observed within the cyst. (**E**, **F**) Axillary lymph node findings, HE stained at ×20, 100 magnification: malignant cells are identified; a scar is seen on the right side. HE, hematoxylin and eosin; pCR, pathological complete response

The histopathological results revealed a pCR with no viable tumor cells in the breast or lymph nodes. There was evidence of necrosis in 1 lymph node, consistent with prior tumor involvement (**Fig. 12**).

As postoperative treatment, radiotherapy was administered to the chest wall and clavicular region starting July 20XX. In addition, pembrolizumab (200 mg) was resumed for 9 cycles, completed in August 20XX. After 21 months, the patient remains disease-free, with no evidence of local recurrence or distant metastases.

## DISCUSSION

The use of NAC is standard for most patients diagnosed with locally advanced TNBC.^1)^ Preoperative chemotherapy, including ICIs, has been widely accepted as the most likely regimen to obtain a pCR in TNBC.^2)^ Furthermore, the residual tumor volume is strongly correlated with prognosis and overall survival.^4,5)^ The tumor’s large size and findings of necrosis initially raised concerns about the efficacy of ICI therapy.^6,7)^ A meta-analysis of the available literature indicated that the efficacy of NAC for TNBC was higher in cases with a smaller tumor size, younger age, lower clinical stage, and absence of lymph node involvement.^8)^ In this case, the tumor was >5 cm in diameter; however, the regimen was remarkably effective.

An increase in the tumor size due to internal necrotic components or inflammation in the surrounding area presents a challenge, as there are no current clinical criteria to determine whether these findings are indicative of cancer progression or changes in response to treatment. Consequently, clinicians must exercise discretion in each case. The clinical findings in this case were characterized by the presence of inflammation due to the breast tumor, accompanied by pain and fever. This case is unique in its presentation, differing from previously reported cases in the response pattern to NAC (concentric shrinkage without or with surrounding lesions, or shrinkage with residual multinodular lesions, or diffuse contrast enhancement in the entire quadrant, or non-visualization), characterized by rapid tumor growth and inflammatory changes during ICI-based therapy.^9,10)^ Also, fever following NAC treatment, including ICIs, can occur for a variety of reasons, including hypopituitarism and cytokine release syndrome, in addition to febrile neutropenia associated with the use of anticancer drugs. In this case, given that other etiologies had been excluded, the decision was made to perform surgery to control the inflammation and pain, regardless of whether the disease was progressing. In this instance, predicting the pCR based on the clinical findings immediately preceding surgery poses a significant challenge. Similarly, identifying a cCR in clinical trials conducted globally to ascertain whether invasive treatment can be circumvented in patients with TNBC following preoperative chemotherapy is a difficult task.^11,12)^ This case highlights the challenges of accurately identifying pCR in clinical practice. In this clinical case, the changes to the tumor caused by preoperative chemotherapy, which included ICI, complicated the assessment of the tumor's extent and the selection of an appropriate treatment plan. Further studies are warranted to clarify the mechanisms driving these responses and optimize management strategies.

## CONCLUSIONS

The evaluation and management of inflammatory responses during immunotherapy represent a critical topic for future research. The insights gained from this case may serve as the foundation for novel therapeutic approaches in TNBC.

## ACKNOWLEDGMENTS

We thank the patient and her family for their cooperation and support throughout the treatment process.

## DECLARATIONS

### Funding

No specific funding was received for this case report.

### Authors’ contributions

K.G.: Data curation and writing—original draft.

To.Y.: Writing—review and editing.

M.Y., Ta.Y., S.F., A.T., E.Y., and Y.Y.: Investigation.

All authors have read and approved the final manuscript.

All authors agree to be responsible for all aspects of the study.

### Ethics approval and consent to participate

Not applicable.

### Consent for publication

Written informed consent was obtained from the patient for publication of this case report and accompanying images.

### Competing interests

The authors declare no competing interests.
